# Immunogenicity of Yellow Fever Vaccine Coadministered With MenAfriVac in Healthy Infants in Ghana and Mali

**DOI:** 10.1093/cid/civ603

**Published:** 2015-11-09

**Authors:** Panchali Roy Chowdhury, Christian Meier, Hewad Laraway, Yuxiao Tang, Abraham Hodgson, Samba O. Sow, Godwin C. Enwere, Brian D. Plikaytis, Prasad S. Kulkarni, Marie-Pierre Preziosi, Matthias Niedrig

**Affiliations:** 1 Centre for Biological Threats and Special Pathogens, Robert Koch Institut, Berlin, Germany; 2 Meningitis Vaccine Project, PATH, Seattle, Washington; 3 Navrongo Health Research Centre, Ghana Health Service, Navrongo, Ghana; 4 Centre pour le Développement des Vaccins, Ministère de la Santé, Bamako, Mali; 5 Meningitis Vaccine Project, PATH, Ferney-Voltaire, France; 6 Centers for Disease Control and Prevention, Atlanta, Georgia; 7 Serum Institute of India Limited, Pune; 8 Meningitis Vaccine Project, Department of Immunization, Vaccines and Biologicals, World Health Organization, Geneva, Switzerland

**Keywords:** yellow fever vaccine, group A meningococcal conjugate vaccine, coadministration, microneutralization assay, Africa

## Abstract

***Background.*** Yellow fever (YF) is still a major public health problem in endemic regions of Africa and South America. In Africa, one of the main control strategies is routine vaccination within the Expanded Programme on Immunization (EPI). A new meningococcal A conjugate vaccine (PsA-TT) is about to be introduced in the EPI of countries in the African meningitis belt, and this study reports on the immunogenicity of the YF-17D vaccines in infants when administered concomitantly with measles vaccine and PsA-TT.

***Methods.*** Two clinical studies were conducted in Ghana and in Mali among infants who received PsA-TT concomitantly with measles and YF vaccines at 9 months of age. YF neutralizing antibody titers were measured using a microneutralization assay.

***Results.*** In both studies, the PsA-TT did not adversely affect the immune response to the concomitantly administered YF vaccine at the age of 9 months. The magnitude of the immune response was different between the 2 studies, with higher seroconversion and seroprotection rates found in Mali vs Ghana.

***Conclusions.*** Immunogenicity to YF vaccine is unaffected when coadministered with PsA-TT at 9 months of age. Further studies are warranted to better understand the determinants of the immune response to YF vaccine in infancy.

***Clinical Trials Registration.*** ISRCTN82484612 (PsA-TT-004); PACTR201110000328305 (PsA-TT-007).

Yellow fever (YF), an acute viral hemorrhagic fever caused by yellow fever virus, remains among the most feared diseases. Primary endemic regions for YF are sub-Saharan Africa, Central America, and South America. At present, the disease still affects approximately 200 000 persons with 30 000 deaths annually, despite the availability of YF vaccines. YF vaccines are live attenuated vaccines based on the 17D attenuation variant and are considered among the most effective and safe vaccines in use today, with >400 million people vaccinated [1–6].

One of the main public health concerns is the maintenance of high levels of population immunity in endemic regions through routine childhood immunization. The YF-17D vaccine was added to the Expanded Programme on Immunization (EPI) in YF-endemic countries in Africa in 1991, in concomitant administration with measles vaccine in infants at 9 months of age [7]. However, there are still only limited data available on safety and immunogenicity when YF-17D vaccine is coadministered with other vaccines. The immune response is usually not affected when coadministered with other vaccines such as measles [8, 9]; however, some reports have described significantly lower immune responses to YF, mumps, and rubella following coadministration of YF and measles, mumps, and rubella vaccine [10].

A monovalent group A meningococcal conjugate vaccine (PsA-TT, MenAfriVac), developed through the Meningitis Vaccine Project (MVP), is about to be introduced in routine EPI in countries of the African “meningitis belt,” with a single dose at 9 months of age concomitantly administered with YF and measles vaccines [11]. We report here the immune response to YF vaccine following coadministration with PsA-TT in 2 infant clinical trials conducted in Ghana and in Mali.

## METHODS

The studies were designed and conducted in accordance with the Good Clinical Practice guidelines established by the International Conference on Harmonisation, and with the Declaration of Helsinki, and approved by the competent ethics committees and regulatory authorities. Both studies were coordinated by MVP, a partnership between the World Health Organization (WHO) and PATH, aiming to develop an affordable, monovalent, group A meningococcal conjugate vaccine through a public–private partnership with the vaccine manufacturer Serum Institute of India, Ltd.

### Study A

The first study (PsA-TT-004) was a phase 2, double-blind, randomized, controlled, dose-ranging study to evaluate the safety, immunogenicity, dose response, and schedule response of PsA-TT administered concomitantly with local EPI vaccines in healthy infants. The study was conducted in rural northern Ghana from November 2008 to May 2012 and the main study results are reported by Hodgson et al (unpublished data). A total of 1200 infants were randomized to receive primary vaccination into 6 study groups of 200 subjects each. Subjects’ group allocation during the study is presented in Table 1. Subjects in all groups received EPI vaccines (measles and YF) at 9 months of age. The EPI vaccines were administered alone in groups 3 and 4, and concomitantly with a second dose of PsA-TT with different dosages in groups 1A (10 µg), 1B (5 µg), and 1C (2.5 µg) and with a single dose of PsA-TT (10 µg) in group 2. Group 4 was the control group for this vaccine period (no blood draw was performed in group 3 at this time point).

**Table 1.** Summary Description of 2 Infant Studies and Demographics of Study Subjects at Yellow Fever Vaccination

Study ID	Study Site	No. of Subjects Enrolled/No. of Subjects by Study Arm	Study Group	Vaccines Administered by Study Arm				
				At Age 14–18 wk: DTwPHBVHib – OPV in All Study Groups	At Age 9–12 mo: Measles–YF in All Study Groups	At Age 12–18 mo: DTwPHBVHib in All Study Groups	At Age 15–18 mo: Measles/Rubella in All Study Groups	No. of Subjects at YF Vaccination, Sex: F/M, Age, Median (Min-Max)
Study A: PsA-TT-004	Navrongo, Ghana	1200/ 200 per group	1A	PsA-TT 10 µg	PsA-TT 10 µg	…	NA	
			1B	PsA-TT 5 µg	PsA-TT 5 µg	…		1153
			1C	PsA-TT 2.5 µg	PsA-TT 2.5 µg	…		573/580
			2	…	PsA-TT 10 µg	…		9 mo
			3	…	…	PsA-TT 10 µg		(8–13 mo)
			4	…	…	…		
Study B: PsA-TT-007	Bamako, Mali	1500/ 300 per group	1A	NA	PsA-TT 10 µg	NA	PsA-TT 10 µg	
			1B		PsA-TT 5 µg		PsA-TT 5 µg	1500
			2A		PsA-TT 10 µg		…	725/775
			2B		PsA-TT 5 µg		…	9 mo
			3		…		…	(9–13 mo)

Abbreviations: DTwPHBVHib, diphtheria, tetanus, whole-cell pertussis (DTwP), hepatitis B virus (HBV), *Haemophilus influenzae* type b (Hib) vaccines; NA, not applicable; OPV, oral polio vaccine; PsA-TT, group A meningococcal conjugate vaccine; YF, yellow fever.

### Study B

The second study (PsA-TT-007) was a phase 3, double-blind, randomized controlled study to evaluate the immunogenicity and safety of different schedules and formulations of PsA-TT administered concomitantly with local EPI vaccines in healthy infants and toddlers. The study was conducted in urban Mali from March 2012 to September 2013, and the main study results are reported by Hodgson et al (unpublished data). A total of 1500 infants were randomized to receive primary vaccination into 5 study groups of 300 subjects each. Subjects’ group allocation during the study is presented in Table 1. Subjects in all groups received EPI vaccines (measles and YF) at 9 months of age. The EPI vaccines were administered alone in group 3, and concomitantly with PsA-TT vaccine with different dosages in groups 1A (10 µg), 1B (5 µg), 2A (10 µg), and 2B (5 µg). Group 3 was the control group for this vaccine period.

### Yellow Fever Vaccines

#### Study A

The live attenuated YF virus vaccine strain 17D, substrain 17DD (Fiocruz Yellow Fever Vaccine, manufactured by Bio-Manguinhos/Fiocruz) was used. The vaccine contained ≥1000 LD_50_ (lethal dose, 50%) units per dose (0.5 mL); that is, the vaccine concentration per dose was between 4.34 log_10_ plaque-forming units (PFU) and 4.56 log_10_ PFU (2 batches, No. 085VFA051Z and No. 085UFC011Z, were used). The presentation was in 10-dose vials of freeze-dried vaccine to be reconstituted with diluent.

#### Study B

The live attenuated YF virus vaccine strain 17D (manufactured by Federal State Unitary Enterprise of Chumakov Institute of Poliomyelitis and Viral Encephalitis, Russian Academy of Medical Sciences) was used. This vaccine contains ≥1000 LD_50_ units per dose (0.5 mL); that is, the vaccine concentration per dose was between 4.5 log_10_ PFU and 4.7 log_10_ PFU (a single batch was used, No. 090). The presentation was in 5-dose vials of freeze-dried vaccine to be reconstituted with diluent.

### Immunogenicity

Blood samples obtained before and 4 weeks after YF vaccination were tested for neutralizing antibodies against YF virus in the Robert Koch Institute (RKI) microneutralization assay using the YF-17D target virus strain produced at the RKI in a concentration of 100 TCID_50_ (tissue culture infectious dose, 50%)/well (ie, 100 µL) [12]. Neutralization titers (NTs) were expressed as the reciprocal serum dilutions yielding ≥50% neutralization after 5 days, that is, blocking at least 1 of 2 duplicate infections.

All serum samples were first heat-inactivated at 56°C for 30 minutes; then, 2-fold dilutions of each serum sample were prepared in 96-well plates to obtain dilutions of 1:4 to 1:256. To each serum dilution the same volume of YF-17D virus was added. The serum-virus mixture along with positive and negative control sera was incubated for 1 hour at 37°C in a 5% carbon dioxide, 90% humidity atmosphere. Meanwhile, porcine kidney epithelial (PS) cells (10 mL of 6 × 10^5^ cells/mL) were prepared. PS cells were washed with phosphate-buffered saline, detached by the addition of HyQTase (incubation for 10 minutes at 37°C), and then diluted in Dulbecco's modified Eagle medium to the required concentration. A volume of 100 µL of the correctly adjusted cell concentration was then added to each well of a new 96-well plate. After 1 hour of incubation, 100 µL of each serum–virus solution was transferred in duplicates to the wells with the cells. For each serum sample, cytotoxicity (to exclude possible cytotoxic effects of the serum on the cells), cell, and virus controls were used. The plates were incubated for 5 days at 37°C in a 5% carbon dioxide, 90% humidity atmosphere, and then cells were fixated with 3.7% formaldehyde and stained with naphthalene black solution. Plates were evaluated and each well was observed under a microscope for signs of cytopathic effects in the infected cells. The serum dilution, which prevented 50% of replicate inoculation (ie, in which 1 of 2 duplicate infections was blocked), was determined as the NT. Whenever infection was prevented in both duplicate wells (100%) at a particular dilution and present in both duplicates (100%) at the next dilution, the NT was determined as the geometric mean of the 2 dilutions. If complete infection was observed at all serum dilutions, the NT was determined as <1:4 the starting serum dilution.

Seroconversion was defined as an NT at least twice as high as that at baseline (≥2-fold rise) 28 days after immunization. Seroprotection was defined as an NT ≥1:8.

### Statistical Analysis

The neutralizing geometric mean titers (GMTs) between the vaccine groups at baseline and 4 weeks after vaccination were compared using analysis of variance (ANOVA) adjusted for baseline titers, age, and sex. Percentages of subjects with NTs ≥2-fold rise and with NTs ≥1:8, along with their exact binomial 95% confidence interval (CI), were calculated. The 95% CI for the difference in the proportions of subjects with these responses between the control group and a particular study vaccine group where subjects received PsA-TT was computed using the Miettinen–Nurminen method [13]. If the upper limit of the CI was <10%, the response in the study vaccine group was considered to be noninferior to that of the control group. Reverse cumulative distribution curves of YF NTs were generated at baseline prior to vaccination and 4 weeks after vaccination. All immunogenicity analyses were conducted in the intention-to-treat population. Missing values were treated as missing at random. All tests were 2-sided with a significance level of .05. Data analysis was performed using SAS, version 9.1.3.

## RESULTS

### Study A

#### Study Population

A total of 1153 subjects (96% of the 1200 subjects enrolled at age 14 weeks) received YF vaccination at a median age of 9 months with a sex ratio (F/M) of 0.99. The immune response to YF vaccine was assessed in all study subjects with sufficient volumes of sera (Table 2).

**Table 2.** Summary of Outcomes of Yellow Fever Neutralizing Antibody Titers in 2 Infant Studies, at 28 Days After Vaccination

Age	Study ID	Vaccine Group	No. Tested^a^/Vaccinated With Yellow Fever Vaccine	Yellow Fever NT Titer ≥1:8, % (95% CI)	Yellow Fever NT ≥2-Fold Rise, % (95% CI)	Yellow Fever NT, Geometric Mean Titer (95% CI)
9 mo	Study A^b^: PsA-TT-004	Group 1A PsA-TT 10 µg (dose 2)	174/193	79.3 (72.5–85.1)	68.4 (60.9–75.2)	16.6 (13.9–19.9)
		Group 1B PsA-TT 5 µg (dose 2)	162/191	74.7 (67.3–81.2)	71.0 (63.3–77.8)	15.0 (12.4–18.2)
		Group 1C PsA-TT 2.5 µg (dose 2)	177/194	67.8 (60.4–74.6)	64.8 (57.2–71.8)	12.1 (10.1–14.5)
		Group 2 PsA-TT 10 µg (dose 1)	160/189	70.0 (62.3–77.0)	67.1 (59.2–74.3)	12.6 (10.5–15.1)
		Group 3 Control	…	…		
		Group 4 Control	168/190	71.4 (64.0–78.1)	67.1 (59.4–74.1)	15.2 (12.5–18.6)
	Study B^c^: PsA-TT-007	Group 1A PsA-TT 10 µg (dose 1)	60/300	98.3 (91.1–100)	98.3 (91.1–100)	33.9 (28.9–39.7)
		Group 1B PsA-TT 5 µg (dose 1)	60/300	96.7 (88.5–99.6)	91.7 (81.6–97.2)	33.3 (27.5–40.3)
		Group 2A PsA-TT 10 µg (dose 1)	61/300	95.1 (86.3–99.0)	93.4 (84.1–98.2)	32.5 (26.8–39.4)
		Group 2B PsA-TT 5 µg (dose 1)	59/300	96.6 (88.3–99.6)	89.8 (79.2–96.2)	31.6 (25.8–38.7)
		Group 3 Control	60/300	96.7 (88.5–99.6)	90.0 (79.5–96.2)	29.1 (24.6–34.6)

Abbreviations: CI, confidence interval; NT, neutralization titers; PsA-TT, group A meningococcal conjugate vaccine.

^a^ No. of subjects tested at 28 days after vaccination.

^b^ Study A: The difference in ≥1:8 percentage was −7.9% (95% CI, −17.0 to 1.3) between group 4 and group 1A, −3.3% (95% CI, −12.8 to 6.4) between group 4 and group 1B, 3.6% (95% CI, −6.1 to 13.3) between group 4 and group 1C, and 1.4% (95% CI, −8.4 to 11.3) between group 4 and group 2; the difference in ≥2-fold rise percentage was −1.3% (95% CI, −11.2 to 8.6) between group 4 and group 1A, −3.9% (95% CI, −13.9 to 6.1) between group 4 and group 1B, 2.3% (95% CI, −7.8 to 12.3) between group 4 and group 1C, and −0.0% (95% CI, −10.2 to 10.2) between group 4 and group 2, 4 weeks after vaccination by Miettinen–Nurminen method. For the comparison of NT geometric mean titers (GMTs) between groups, the *P* value was >.05 (all groups) by analysis of variance (ANOVA) after adjusting for age, sex, and baseline titer.

^c^ Study B: The difference in ≥1:8 percentage was −1.7% (95% CI, −10.0 to 5.9) between group 3 and group 1A, 0.0% (95% CI, −8.5 to 8.5) between group 3 and group 1B, 1.6% (95% CI, −7.1 to 10.7) between group 3 and group 2A, and 0.1% (95% CI, −8.5 to 8.7) between group 3 and group 2B; The difference in ≥2-fold rise percentage was −8.3% (95% CI, −18.8 to .1) between group 3 and group 1A, −1.7% (95% CI, −13.1 to 9.6) between group 3 and group 1B, −3.4% (95% CI, −14.6 to 7.2) between group 3 and group 2A, and 0.2% (95% CI, −11.5 to 12.0) between group 3 and group 2B, 4 weeks after vaccination by Miettinen–Nurminen method. For the comparison of NT GMTs between groups, the *P* value was >.05 (all groups) by ANOVA after adjusting for age, sex, and baseline titer.

#### YF Serum Neutralizing Antibody Titers

**Figure 1.**
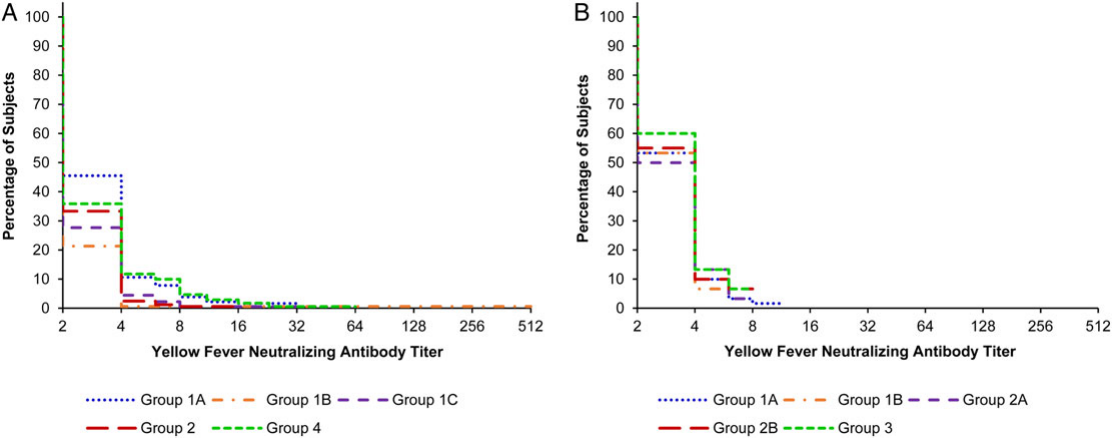
Reverse cumulative distribution curves for yellow fever neutralizing antibody titers in studies A (*A*) and B (*B*), prior to vaccination at 9 months of age and according to the vaccine group.

**Figure 2.**
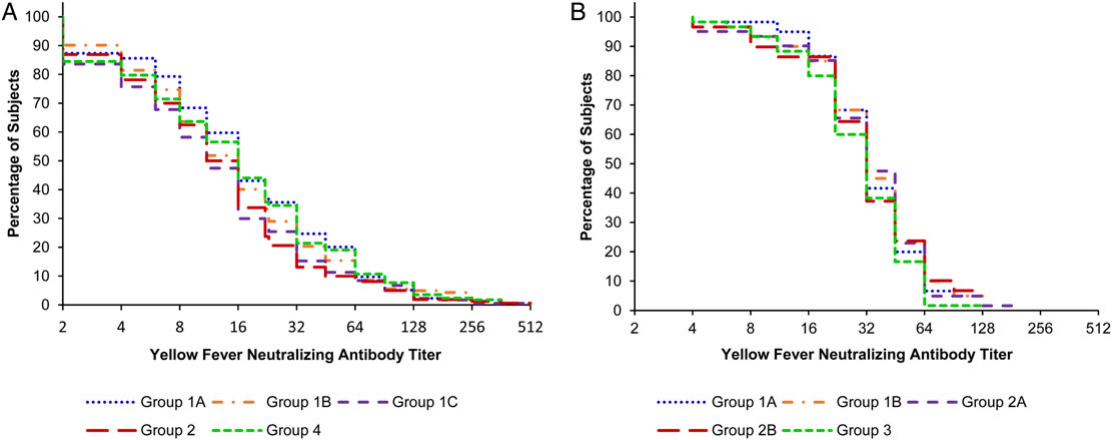
Reverse cumulative distribution curves for yellow fever neutralizing antibody titers in studies A (*A*) and B (*B*), at 4 weeks after vaccination and according to the vaccine group.

Reverse cumulative distribution curves for YF NTs at baseline prior to vaccination and 4 weeks after vaccination, according to study groups, are shown in Figures 1*A* and 2*A*. The proportion of subjects with YF NTs ≥1:8, with ≥2-fold YF NT rises as compared to baseline and the GMTs of YF fever NTs, at 28 days after vaccination and for each study group, is presented in Table 2.

##### At Baseline

As shown in Figure 1*A*, a handful of subjects had YF titers ≥1:8. Overall, the percentage of subjects with YF titers ≥1:8 was 4.5% (38/851), ranging from 0.6% (95% CI, .07%–3.4%) to 10.0% (95% CI, 5.9%–15.5%), in the vaccine groups. The GMTs of YF titers ranged from 2.4 (95% CI, 2.2–2.6) to 3.0 (95% CI, 2.8–3.3).

##### At 28 Days After Vaccination

The percentages of subjects with YF titers ≥1:8 ranged from 67.8% to 79.3% and were lower than the anticipated response rate (95%) in all groups. The noninferiority of the immune response elicited by YF vaccine administered concomitantly with the second dose of PsA-TT vaccine at different dosages (10 µg and 5 µg) to that elicited by YF vaccine alone was demonstrated—that is, the upper limits of the 95% CI for the differences were <10% between group 4 and each of groups 1A and 1B. In contrast, the same noninferiority was not confirmed when YF vaccine was administered concomitantly with the second dose of PsA-TT 2.5 µg vaccine or with the 1-dose 10 µg PsA-TT vaccine, with an upper limit of the CI of the difference of 13.3% between group 4 and group 1C and of 11.3% between group 4 and group 2, respectively (Table 2).

The percentages of subjects with a ≥2-fold response in YF titers with respect to baseline ranged from 64.8% to 71.0%. The noninferiority of the immune response elicited by YF vaccine administered concomitantly with the second dose of PsA-TT vaccine at different dosages (10 µg and 5 µg) to that elicited by EPI vaccines alone at 28 days after vaccine administration was demonstrated for this endpoint as well; the upper limit of the 95% CI for the differences was <10% between group 4 and each of groups 1A and 1B. In contrast, the same noninferiority was not confirmed when YF vaccine was administered concomitantly with the second dose of PsA-TT 2.5 µg vaccine or with the 1-dose 10 µg PsA-TT vaccine; that is, the upper limit of the 95% CI for the differences was 12.3% between group 4 and group 1C and 10.2% between group 4 and group 2, respectively (Table 2).

YF neutralizing GMTs were similar in all groups, ranging from 12.1 to 16.6, with no statistically significant difference when groups were compared using ANOVA after adjusting for age, sex, and baseline titer (Table 2), and the distribution of YF NTs was consistently similar in all study groups (Figure 2*A*).

Four weeks postvaccination, analysis stratified by sex did not show any difference in the overall proportion of subjects with YF NTs ≥1:8 (72.6% [95% CI, 68.0%–76.9%] among girls and 72.7% [95% CI, 68.2%–76.8%] among boys) or with ≥2-fold YF neutralizing titer rises (68.8% [95% CI, 64.1%–73.3%] among girls and 66.5% [95% CI, 61.8%–71.0%] among boys), or in the overall GMTs of YF fever neutralizing titers (14.0 [95% CI, 12.4–15.7] among girls and 14.4 [95% CI, 12.8–16.2] among boys).

### Study B

#### Study Population

All 1500 subjects enrolled received YF vaccination at a median age of 9 months, with a sex ratio (F/M) of 0.94. The immune response to YF vaccine was assessed in a random subsample of 300 subjects with equal distribution in all study groups (60 subjects per group).

#### YF Serum Neutralizing Antibody Titers

Reverse cumulative distribution curves for YF NTs at baseline prior to vaccination and 4 weeks after vaccination, according to study groups, are shown in Figures 1*B* and 2*B*. The proportion of subjects with YF NTs ≥1:8, with ≥2-fold YF NT rises as compared to baseline and the GMTs of YF fever NTs, at 28 days after vaccination and for each study group, is presented in Table 2.

##### At Baseline

The YF NT at baseline prior to vaccination at 9 months of age was consistently low in all study groups (Figure 1*B*). The percentages of subjects with YF titers ≥1:8 was 4.0% overall (12/300), ranging from 0.0% (95% CI, .0%–4.9%) to 6.7% (95% CI, 1.8%–16.2%) in the different study groups. The GMTs of YF titers ranged from 3.0 (95% CI, 2.7–3.3) in group 1B to 3.3 (95% CI, 2.9–3.7) in group 3.

##### At 28 Days After Vaccination

The percentages of subjects with YF titers ≥1:8 were similar in all groups, ranging from 95.1% to 98.3%, and above the anticipated response rate (95%). The noninferiority of the immune response elicited by YF vaccine administered concomitantly with PsA-TT at different dosages (10 µg and 5 µg) to that elicited by YF vaccine alone was demonstrated; that is, the upper limits of the 95% CI for the differences were <10% between group 3 and each of groups 1A, 1B, and 2B. However, the same noninferiority of group 2A (YF and PsA-TT 10 µg vaccines) to group 3 (YF vaccine alone) was not confirmed with respect to the same endpoint; that is, the upper limit of the 95% CI for the difference was ≥10% (10.7% between group 3 and group 2A) (Table 2).

The percentages of subjects with a ≥2-fold response in YF titer with respect to baseline ranged from 89.8% to 98.3%. The noninferiority of the immune response elicited by YF vaccine administered concomitantly with the first dose of PsA-TT at different dosages (10 µg and 5 µg) to that elicited by YF vaccine alone was demonstrated for this endpoint as well; that is, the upper limit of the 95% CI for the differences was <10% for each comparison of group 3 with groups 1A, 1B, and 2A. However, the same noninferiority of group 2B (YF and PsA-TT 5-µg vaccines) to group 3 (YF vaccine alone) was not confirmed with respect to the same endpoint; that is, the upper limit of the 95% CI for the difference was ≥10% (12.0% between group 3 and group 2B; Table 2).

YF neutralizing GMTs were similar in all groups, ranging from 29.1 to 33.9, with no statistically significant difference when groups were compared using ANOVA after adjusting for age, sex, and baseline titer (Table 2); in addition, the distribution of YF NTs was consistently similar in all study groups (Figure 2*B*).

Four weeks postvaccination, analysis stratified by sex did not show any difference in the overall proportion of subjects with YF titers ≥1:8 (94.9% [95% CI, 89.7%–97.9%] and 98.2% [95% CI, 94.8%–99.6%] among girls and boys, respectively) or with ≥2-fold YF NT rises (91.2% [95% CI, 85.1%–95.4%] and 93.9% [95% CI, 89.1%–97.0%] among girls and boys, respectively), or in the overall GMTs of YF fever NTs (29.4 [95% CI, 26.1–33.1] and 34.4 [95% CI, 30.8–38.3] among girls and boys, respectively).

## DISCUSSION

In both studies, PsA-TT (at 10-µg, 5-µg, and 2.5-µg dosages) did not adversely affect the immune response to the concomitantly administered YF vaccine at the age of 9 months.

In both studies, the noninferiority of each PsA-TT vaccine group to the control group (YF/measles vaccines alone) was demonstrated for the majority of pairwise comparisons of percentages of subjects achieving seroconversion and seroprotection 4 weeks after immunization. In a few instances, such noninferiority was not confirmed, likely due to low statistical power, resulting from low seroconversion rates in study A or from small sample size in study B. In study A, 68%–79% of subjects reached YF seroprotection (NT ≥1:8) at 4 weeks after immunization (ie, significantly less than the expected 95%), resulting in a low power in testing noninferiority. In study B, YF endpoints were measured only in a random subsample of subjects (300/1500, 60 subjects per study group), also resulting in limited power. However, there was no statistically significant difference among all study groups in each study in YF virus neutralizing antibody GMTs 4 weeks after immunization after adjusting for age, sex, and prevaccination titer.

The immune response to YF, as measured by NTs 4 weeks after immunization, was different between the 2 studies, with a higher seroconversion rate, seroprotection rate, and GMTs (93%, 97% and 32, respectively, in study B conducted in Mali, vs 68%, 73%, and 14, respectively, in study A conducted in Ghana). Several determinants could explain this difference, such as vaccine substrain, vaccine concentration, presence of maternal antibodies, and interference of other vaccines [14].

Two different vaccine substrains of YF-17D were used in the 2 studies: the 17DD substrain in study A (Ghana) and the 17D-213/77 substrain (a derivate of the 17D-204 substrain) in study B (Mali). The difference between these 2 vaccine substrains is the passage level (17D-204: 235–240; 17DD: 286–287) [15], but there are only minor differences when comparing nucleotide sequences of both the substrains [16]. Camacho et al and Nascimento Silva et al performed studies in which both vaccine substrains, 17DD and 17D-213, were tested for immunogenicity in adults and infants [10, 17], with no significant difference in immune response. Seroconversion rates were 98% in one study and 70%–88% in the other study. Immunogenicity studies of YF-17D vaccines in infants show that immune responses tend to be lower in infants than in adults, with seroconversion rates ranging from 70% to 88.8% [8, 10, 17], consistent with our findings in study A. Interestingly, Nascimento Silva et al have also reported a significant difference in seroconversion rates when administering YF-17D alone or simultaneously with measles, mumps, and rubella vaccine (86.5% vs 69.5%) [10]. Similar rates were reported in another study, where 9- to 11-month-old infants received the YF-17D vaccine concomitantly with measles vaccine [14] with a seroconversion rate of 72%, consistent with that found in study A.

The difference in immune response between the 2 studies could also be related to a differential amount of viral particles in the vaccines. In 2009, a WHO expert committee defined a minimum amount of viral particles per dose as 3.0 log_10_ international units, that is, approximately equivalent to 3.73 log_10_ PFU. In 2013, the latter concentration was supported by a dose-response study of the YF-17DD vaccine conducted by Martins et al, who demonstrated that this minimal dose was as immunogenic as higher doses, with little differences in response rates [15]. The concentrations of the vaccines, which were used for both our studies, were above this concentration (study A: 4.34–4.56 log_10_ PFU; study B: 4.5–4.7 log_10_ PFU). However, viral concentrations are determined by titrating the virus on susceptible cells. Most commonly, Vero or PS cells are used for this purpose, with titers being higher when performing the titration on Vero cells vs PS cells (a difference ranging from 0.5 to 1 log_10_). The method for the determination of concentrations is not published and it would be valuable to test both vaccines together on the same cell system.

WHO indicated in 2013 that a single dose of the YF-17D vaccine provides lifelong protective immunity against YF disease and that a booster dose is no longer necessary [18]. This is consistent with the systematic review of the efficacy and duration of immunity after YF vaccination conducted by Gotuzzo et al to assess the need for a booster dose every 10 years [19]. Their findings indicate that, in most studies, seroconversion rates following YF vaccination were >90% and remained >75% several years after immunization. Furthermore, they found some indications that a YF booster dose would only lead to a minor or short-lived increase in neutralizing antibodies due to preexisting antibodies from primary vaccination [19], and they concluded that a YF booster dose would not be needed. Given the rather low neutralizing GMTs found after vaccination in study A, the question may arise whether these titer values are maintained throughout life. Conducting a serosurvey in these infants in 3–5 years would be warranted to evaluate whether titers are maintained or decline with time [18, 19].

The presence of maternal antibodies also plays a role in the immunologic response in infants [20, 21]. The median age at vaccination (9 months) and the prevaccination titers were similar and consistently low in both studies, with 4.5% and 4.0% of the infants with titers ≥1:8 prior to vaccination. Therefore, presence of maternal antibodies cannot explain the different levels of response observed in our 2 studies.

Sex differences in response to YF vaccine have been reported, with contradictory reports of higher responses either among adult males or among adult females [22, 23]. No difference in the immune response to YF vaccine according to sex was found in our studies, so more studies are required to analyze sex differences in immune responses to the YF-17D vaccine in infants.

In conclusion, concomitant administration of the PsA-TT does not affect the response to YF vaccine in African infants. Differences in the postvaccination seroconversion and seroprotection rates in the 2 studies were observed, confirming the need to further document the immune response to YF-17D vaccine in infants.
